# Identification of In Vitro Metabolites of Amoxicillin in Human Liver Microsomes by LC–ESI/MS

**DOI:** 10.1007/s10337-014-2648-2

**Published:** 2014-03-22

**Authors:** Malgorzata Szultka, Rafal Krzeminski, Marek Jackowski, Boguslaw Buszewski

**Affiliations:** 1 Department of Environmental Chemistry and Bioanalytics, Faculty of Chemistry, Nicolaus Copernicus University, Gagarin 7 Street, 87-100 Toruń, Poland; 2 Department of Surgery, Collegium Medicum, Nicolaus Copernicus University, St. Joseph Street 53-59, 85-067 Bydgoszcz, Poland

**Keywords:** High-performance liquid chromatography, Mass spectrometry, Antibiotic drug, Human liver microsomes

## Abstract

Amoxicillin (AMOX) metabolism in human liver microsomes was studied in vitro using liquid chromatography–mass spectrometry (LC/MS). Amoxicillin was incubated with human liver microsomes along with NADPH, and the reaction mixture was analyzed by LC/MS to obtain the specific metabolic profile of the studied antibiotic drug. Positive electrospray ionization was employed as the ionization source. An ACE C18-column (4.6 mm × 150 mm, 3 μm) was implemented with acetonitrile and water (+0.1 % formic acid) in isocratic mode as the mobile phase at the flow 0.4 mL min^−1^. The chemical structures of metabolites were proposed on the basis of the accurate mass measurement of the protonated molecule as well as their main product. Six phase I and one phase II metabolites were detected and structurally described. The metabolism of AMOX occurred via oxidation, hydroxylation and oxidative deamination, as well as through combination of these reactions. Compound M7, with glucuronic acid was also observed as phase II metabolite. Neither sulfate nor glutathione conjugates were detected. This study presents novel information about the chemical structure of the potential AMOX metabolites and provides vital data for further pharmacokinetic and in vivo metabolism studies.

## Introduction

Modern medicine is facing growing difficulties in treatment of bacterial infections resulting from an increasing number of antibiotic-resistant bacterial strains. The cause of this problem is the excessive use or misuse of antibacterial medicines not only in the treatment of human infections, but also in the food industry, veterinary medicine and agriculture. Therefore, the current standard treatment is limited to using of one type of drug against specific pathogens [1]. Amoxicillin, 6-{[amino(4-hydroxyphenyl)acetyl]amino}-3,3-dimethyl-7-oxo-4-thial-1-azabicyclo[3.2.0]heptanes-2-carboxylic acid, belongs to β-lactam antibiotics of penicillin group [2]. This antibiotic acts against Gram-positive and Gram-negative bacteria and is most effective against anaerobes [2–4]. Amoxicillin shows bactericidal effect as it blocks the activity of enzymes involved in synthesis of the cell wall. Bacteria lacking a stiff cell wall are more easily degraded under the influence of autolytic enzymes [5]. The antibiotic is used to treat certain bacterial infections such as gonorrhea, bronchitis, infections of the skin or soft tissue and infections of the upper and lower respiratory tract [2, 4].

Identification of the chemical structure of principal phase I and II metabolites is a basic step to assess the fate of biologically active compounds in an organism [6]. In vitro incubation of the drug with human liver subcellular fractions (e.g., microsomes or the S9 fraction, etc.) is a simple and cost-effective method to acquire initial data regarding its metabolism (biotransformation) [7]. Drug metabolism and pharmacokinetic properties of new chemical entities are extensively investigated at various stages of drug discovery and development. In particular, drug metabolism is important in that it reveals the pharmacological activity and toxicological implications of a drug to ensure that it can be used safely in humans. Based on in vitro metabolism profiles, in vivo metabolism studies are further conducted through the advanced preclinical and phase I/II clinical trials [8–12].

High-performance liquid chromatography coupled with mass spectrometry represents one of the progressive analytical tools to study drug metabolism [13]. Nowadays, mass spectrometers offer high resolution, sensitivity and accurate mass measurement for both precursor and product ions. Therefore, such instrumentation is capable of acquiring data necessary for structure identification of unknown analytes (metabolites) within short time period and is a state-of-the-art technique for drug metabolism studies [14]. Determination of antibiotic drugs (and their potential metabolites) in complex matrices such as aqueous solutions or physiological fluids (plasma, whole blood) requires appropriate isolation and preconcentration methods. In qualitative analyses and laboratory practice in life chemistry (bioanalytics, clinical analysis or medicine), the right selection of susceptible and reproducible methods supporting final determination and validation is essential [13, 14].

In this study, we applied LC/MS to identify phase I and phase II metabolites of AMOX as a model compound detected after in vitro incubation of the studied drug with human liver microsomes fraction. To the best of our knowledge, no detailed information regarding the structure of AMOX metabolites via in vitro studies has been published. Transformations were investigated with HLM and in the presence of NADPH-P450 oxidoreductase. The results obtained will allow prediction of metabolic pathways of AMOX in patients differing in the level of cytochrome P450 isoenzymes and, in turn, can probably be helpful in designing personalized antibacterial treatments including this drug.

## Experimental

### Chemicals

Amoxicillin (AMOX) was supplied by Sigma-Aldrich (Schnelldorf, Germany). Human liver microsomes (HLM), glucose-6-phosphate, β-NADP^+^, glucose-6-phosphate dehydrogenase and uridine 5′-diphosphoglucuronic acid triammonium salt (UDPGA) were obtained from Sigma-Aldrich (Schnelldorf, Germany). Other chemicals of analytical quality (acetonitrile, formic acid) were purchased from Merck (Darmstadt, Germany). Water was obtained by means of Milli-Q RG apparatus by Millipore (Millipore Intertech, Bedford, MA, USA) in our laboratory.

### Phase I In Vitro Incubation

The drug incubation (AMOX, 1–25 μm) with HLM was performed in 100 mM phosphate buffer (PB), pH 7.4, at 37 °C for 90 min in a final reaction mixture volume of 200 μL. The reaction was initiated by the addition of an NADPH-generating system containing 0.8 mM β-NADP^+^, 10 mM glucose-6-phosphate and 1 unit of glucose-6-phosphate dehydrogenase to the reaction mixture. The blank samples were of the same composition as the test samples, but they did not contain AMOX. The omitted component in the control samples was replaced by an appropriate amount of PB to maintain a constant total sample volume. All samples were incubated at 37 °C for 1.5 h and shaken (120 rpm) during the incubation. The reaction was terminated with 50 μl of ice-cold ACN, and the samples were stored at −20 °C. Thereafter, all samples were centrifuged (15,000*g*); then 180 μl of the supernatant was diluted with 220 μL of water, filtered through a 0.22 μm filter and stored at −80 °C until analyzed. All samples were prepared in triplicate.

### Phase II In Vitro Incubation

Glucuronidation was studied by incubation of the model drug with HLM. The test and blank samples were prepared analogously to phase I metabolism samples, the exception being that 10 μL of PB contained the UDPGA (50 mM) co-factor which mediated the relevant reaction. All samples were incubated and treated as described above.

### Instrumentation

The HPLC 1100 (Agilent, Waldbronn, Germany) was used as the chromatographic system. It consisted of quaternary pump (G 1310A), degasser and an automatic sample injection (G 1313A). The chromatographic separation was performed on ACE C18 (150 mm × 4.6 mm i.d., 5 μm particle, 300 Å) column with acetonitrile (+0.1 % formic acid) and water (+0.1 % formic acid) (65:35, v/v) mobile phase at the flow rate of 0.4 mL min^−1^. The column temperature was 21 °C and the injection volume was 15 μL. The chromatographic run time was kept under than 6 min. Additionally, the chromatographic system was coupled to the Agilent 6410 Triple Quad mass spectrometer (Agilent, Waldbronn, Germany) equipped with an electrospray ionization (ESI) interface operated in positive ion mode and with the following operation parameters: gas temperature, 325 °C; gas flow rate, 7.5 L min^−1^; nebulizer gas pressure, 40 psi; and capillary voltage, 3,500 V. Nitrogen was used in the ion source and the collision cell. Full-scan MS and then quadrupole precursor ion selection MS/MS in both positive mode was recorded within the mass range of *m*/*z* 50–600. Additionally, ion acquisition was accomplished in multiple reaction monitoring (MRM) mode for quantification. Agilent Mass Hunter software version B.04.01 was used for data acquisition, instrument control and data analysis. For sample evaporation and centrifugation, a Labconco CentriVap DNA concentrator (Kansas City, USA) was used. Water was purified with a Milli-Q Purification System (Millipore, Bedford, MA, USA).

### Preparation of Stock and Standard Solutions

The working standard drug solutions, based on the therapeutic concentrations, were prepared by diluting the stock solution of amoxicillin (125 μg mL^−1^) to a proper volume. The stock solutions were diluted to make working standard solutions in the range from 1 to 50 μg mL^−1^. The plasma samples were stored at −20 °C. Before use, the plasma was thawed at room temperature and centrifuged at 2,500*g* for 5 min. The spiked human liver microsome samples were prepared so as to reach final concentrations of 1–50 μg mL^−1^.

### Calibration and Validation

The method was validated under optimized conditions. Each calibration curve was the compilation of points covering from 1 to 50 μg mL^−1^. We used calibration curves prepared in triplicate by spiking human blank plasma to obtain the relevant concentration of AMOX. The accuracy was presented as the ratio of the determined and nominal values of the relevant drug concentrations and multiplied by 100 %. In addition, the precision was defined as the percentage of standard deviation of the relevant values divided by the average of mean values. The limit of detection (LOD = 3 × SD_*xy*/*b*_, where SD_*xy*_ is the standard deviation and *b* is the slope) and the limit of quantification (LOQ = 10 × SD_*xy*/*b*_) were calculated with the acceptable precision and accuracy as defined by Konieczka [15]. The recoveries were determined by comparison of the peak areas after HPLC measurements of the spiked samples after respective sample preparation with the direct injection of standard solutions of equal concentrations. Logically, linearity was examined by analyzing blank plasma samples (*n* = 3) fortified with standard solutions of drugs at the same concentrations. The concentration range was obtained with the regression curve (*y* = *ax* + *b*) and correlation coefficient (*R* ^2^). The analytical procedure was fully validated according to the appropriate guidelines of the American Food and Drug Administration [16]. Each point was the average of three injections with the injection volume of 15 μL. Integrated peak areas of the selected quantification MRM transitions were used to build the standard curves. The standard curves were fitted using weighted least square regression analysis (1/χ) utilizing the Quanlynx function based on the signal-to-noise of 3:1 and 10:1, respectively. System control, data acquisition and interpretation were performed with the Agilent Mass Hunter (Version 0.0100) software including the Qualitative package (for chromatographic and spectral interpretation) and the Quantitative software (for quantitative information generation). Calibration curves were set up with the Mass Hunter Quantitative program using non-weighted least squares regression.

### Data Analysis

LC/MS data of the test and blank samples were compared both manually and using relevant software. Post-acquisition analyses were performed using Mass Hunter Metabolite ID (version 01.01) program (Agilent, Waldbronn, Germany), which employs an extensive list of potential biotransformation reaction (e.g., hydroxylation), in combination with the elemental compositions of the substrate molecules, to generate a series of extracted ion chromatograms (XICs). These XICs are compared between the control and sample to eliminate those chromatographic peaks in the sample that also appear in the control. Data mining algorithm “molecular feature extraction” was applied to extract compounds. Additionally, compound mass spectra were generated. The identification was based on accurate mass measurements of precursor and product ions, considering the chemical structure of the parent compound and the general principles of metabolism as well.

## Results and Discussion

### ESI–MS^*n*^ and LC–ESI/MS Studies for Mass Fragmentation Pattern of Amoxicillin

**Fig. 1:**
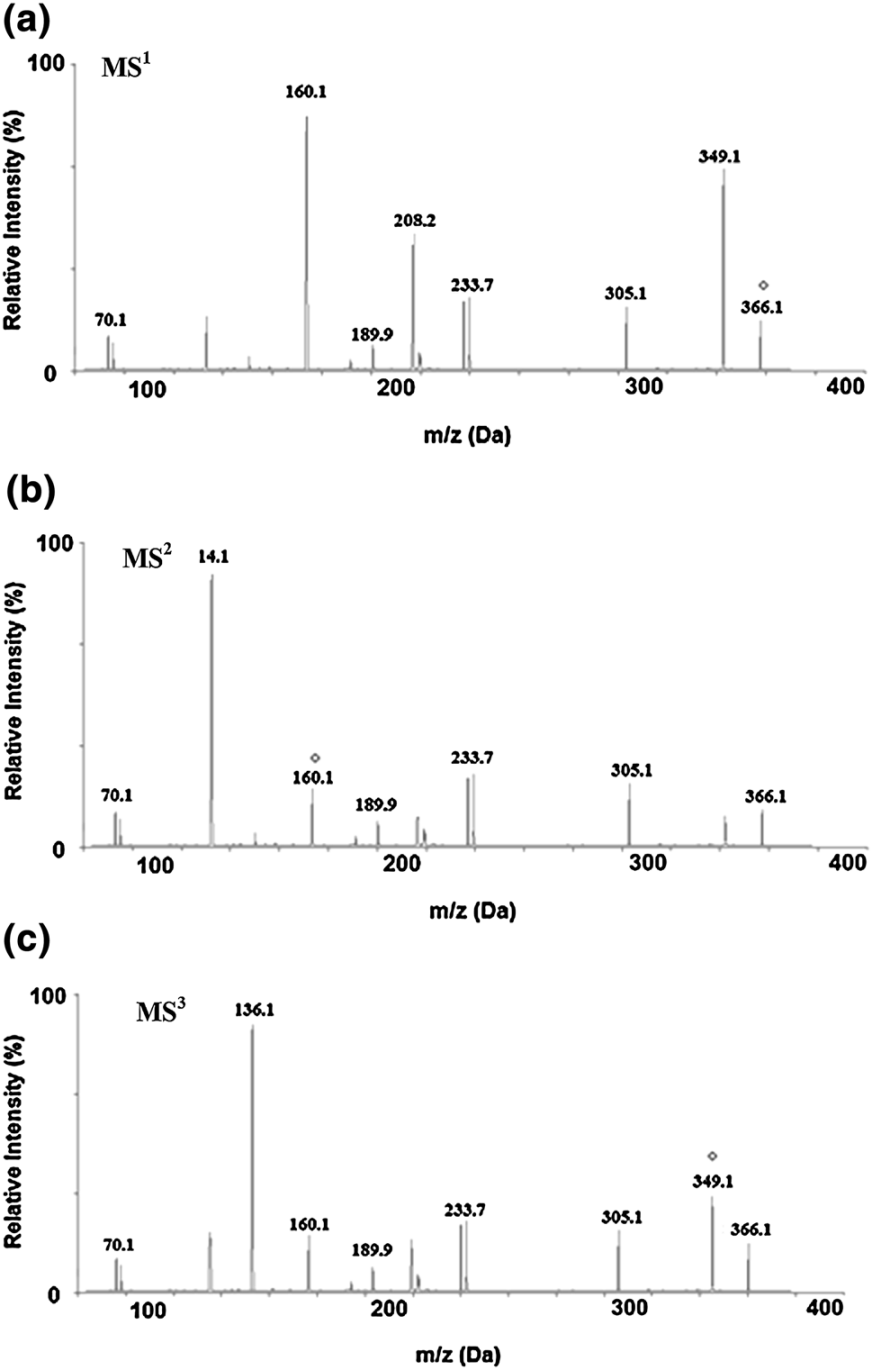
Two-stage mass spectra data of amoxicillin

Two-stage mass spectra (MS^3^) of amoxicillin were recorded to outline its mass fragmentation pattern for assisting the characterization of the fragmentation products (Fig. 1). The drug was detected at *m*/*z* 366 [M+H]^+^ (parent ion) and *m*/*z* 388 [M+Na]^+^ (Na-adduct ion) in the MS^1^ spectrum corresponding to its molecular mass of 365 Da. The fragments of *m*/*z* 233 and 305 were observed both in MS^2^ and MS^3^ spectra. It indicated that they were stabilized probably by the cyclization leading to delocalization of the positive charge and hence became stable against further fragmentation. Fragmentation of the parent ion in MS^1^ produced the heaviest ion (the one with the greatest *m*/*z* value), *m*/*z* 160, which was formed by the loss of 206 Da from *m*/*z* 366. The loss of 158 Da from the ion at *m*/*z* 366 may have led to fragment at *m*/*z* 208. The *m*/*z* 160 was formed in the greatest abundance and thus was targeted as the precursor ion to generate the MS^2^ spectrum that showed product ion at *m*/*z* 114. It was suggested that the *m*/*z* 114 was formed by the loss of 46 Da from *m*/*z* 160. On the basis of the mass and second-highest abundance of the product ion, the *m*/*z* 349, corresponding to the loss of NH_3_, was used as the precursor ion for another MS^3^ study. It mainly fragmented to the product ions of *m*/*z* 305, 233, 189, 136 and 70. The *m*/*z* 136 could have been formed by the loss of 213 Da.

**Fig. 2:**
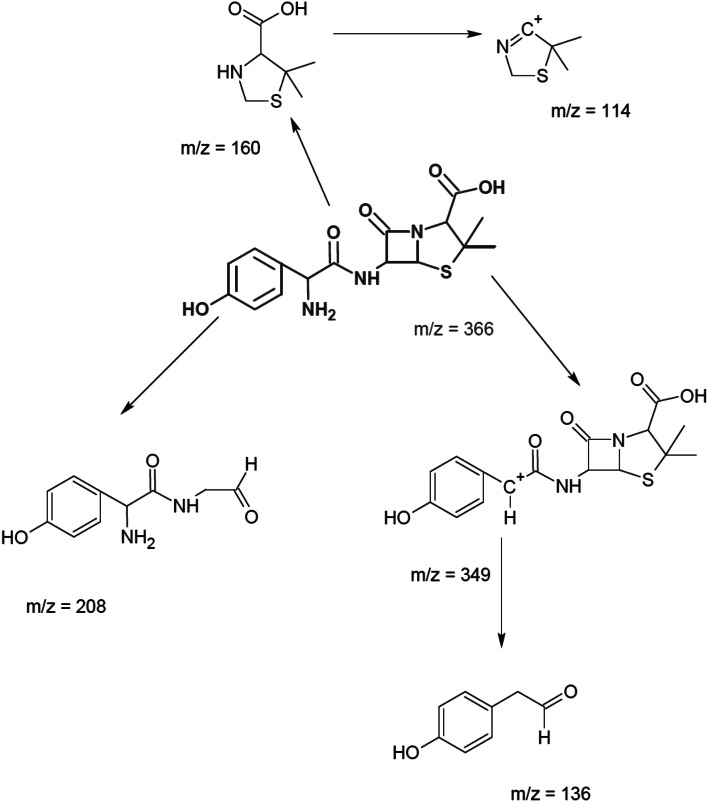
Proposed fragmentation pathway of amoxicillin

The detection of ions by HPLC/ESI/MS/MS made it possible to determine the proposed fragmentation pathway of AMOX, as displayed in Fig. 2. Figure 2 presents the following fragmentation sequence: the loss of 205 Da with the formation of the ion at *m*/*z* 366; the loss of 47 Da with the formation of the ion at *m*/*z* 161; the loss of C_10_H_8_N_2_O_3_ before C_11_H_11_N_2_O_5_ from the ion at *m*/*z* 366 resulting in the ions at *m*/*z* 161 and 114, respectively.

### In Vitro Metabolic Profiling

There are a large number of components in the HLM, many of which may elute during analysis at the same time as the target compounds. To solve this problem, we performed the tests using Mass Hunter Metabolite ID software, and peaks present in the analyte were evaluated with reference to control samples by comparing their retention times during post-acquisition analyses, MS spectra and peak areas.

Under the chromatographic conditions used herein, the parent drug, AMOX, was detected as [M+H] + at *m*/*z* = 366 the retention time of 5.93 min. The fragmentation behavior of the parent drug was studied to provide initial data for the interpretation of mass spectra of its metabolites. The protonated molecule of AMOX gave three main product ions in the MS/MS scan, as mentioned above.

**Fig. 3:**
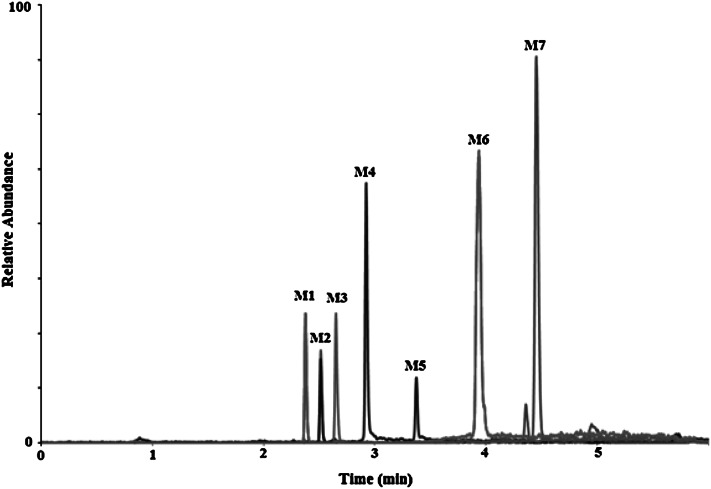
Representative extracted ion chromatogram of amoxicillin and its metabolites produced following the incubation with human liver microsomes

The incubation of amoxicillin with human liver microsomes in the presence of NADPH generated 7 metabolite peaks (S/N ≥ 5), shown in Fig. 3. Extracted compound chromatograms and product ion spectra were derived and analyzed. When AMOX was incubated without microsomes, no metabolites were observed, indicating that all metabolites were generated in the presence of microsomal enzymes. Analysis of the samples incubated with HLM revealed six phase I (M1–M6) metabolites and one phase II metabolite (M7). These potential metabolites were numbered M1–M7 according to their retention time. Additionally, MS/MS experiments using protonated molecules as the precursor ion were performed to confirm the suggested structures of the product ions formed.

**Fig. 4:**
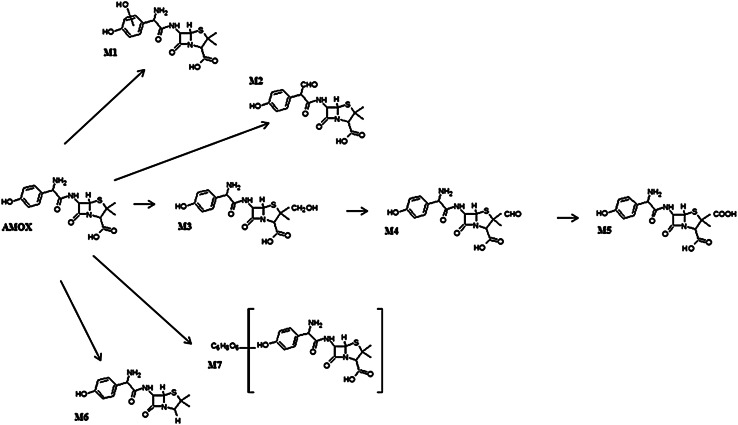
Postulated metabolic pathways of amoxicillin in human liver microsomes

The MH^+^ ions of the metabolites in the HLM samples were identified by full-scan MS detection (Fig. 3; Table 1). The metabolite M1 was observed at *m*/*z* = 382, indicating hydroxylation of the aromatic moiety; M2, at *m*/*z* = 379, indicating oxidative deamination of –NH_2_ group; M3–M5, at *m*/*z* = 382, 380 and 396, respectively, indicating oxidation of aliphatic chain; M6, at *m*/*z* = 322 indicating decarboxylation. The obtained metabolites at *m*/*z* = 379 and 322 are associated with the additional loss of NH_2_ and CO_2_, respectively. Additionally, one glucuronide was detected after incubation of the parent drug with HLM using UDPGA as a co-factor. Metabolite M7 with [M+H]^+^ at *m*/*z* = 542 corresponds to the conjugation of the parent drug with glucuronic acid. Indeed, the MS/MS spectrum showed the typical neutral loss of glucuronic acid (*Δ* −176 Da) and the identical product ion at *m*/*z* = 208 identical with that of the parent drug. Neither sulfate nor glutathione conjugates were detected. The postulated metabolic pathway for all metabolites is presented in Fig. 4. Table 1 shows the retention time, experimental mass *m*/*z*, elemental composition and characteristic metabolic reaction in the MS/MS stage of the target metabolite.

**Table 1:** The retention times, mass characteristics and elemental composition of the [M+H]^+^ of AMOX and the metabolites, M1–M7

Compound	Retention time (min)	[M+H]^+^	Elemental composition	Metabolic reaction
AMOX	5.93	366	C_16_H_20_N_3_O_5_S	–
M1	2.34	382	C_16_H_20_N_3_O_6_S	Hydroxylation
M2	2.52	379	C_17_H_19_N_2_O_7_S	Oxidative deamination
M3	2.76	382	C_16_H_20_N_3_O_6_S	Oxidation of aliphatic chain
M4	2.94	380	C_16_H_18_N_3_O_6_S	Oxidation of aliphatic chain
M5	3.48	396	C_16_H_20_N_3_O_7_S	Oxidation of aliphatic chain
M6	3.98	322	C_15_H_19_N_3_O_3_S	Decarboxylation
M7	4.49	542	C_25_H_28_N_3_O_11_S	Glucuronidation

**Fig. 5:**
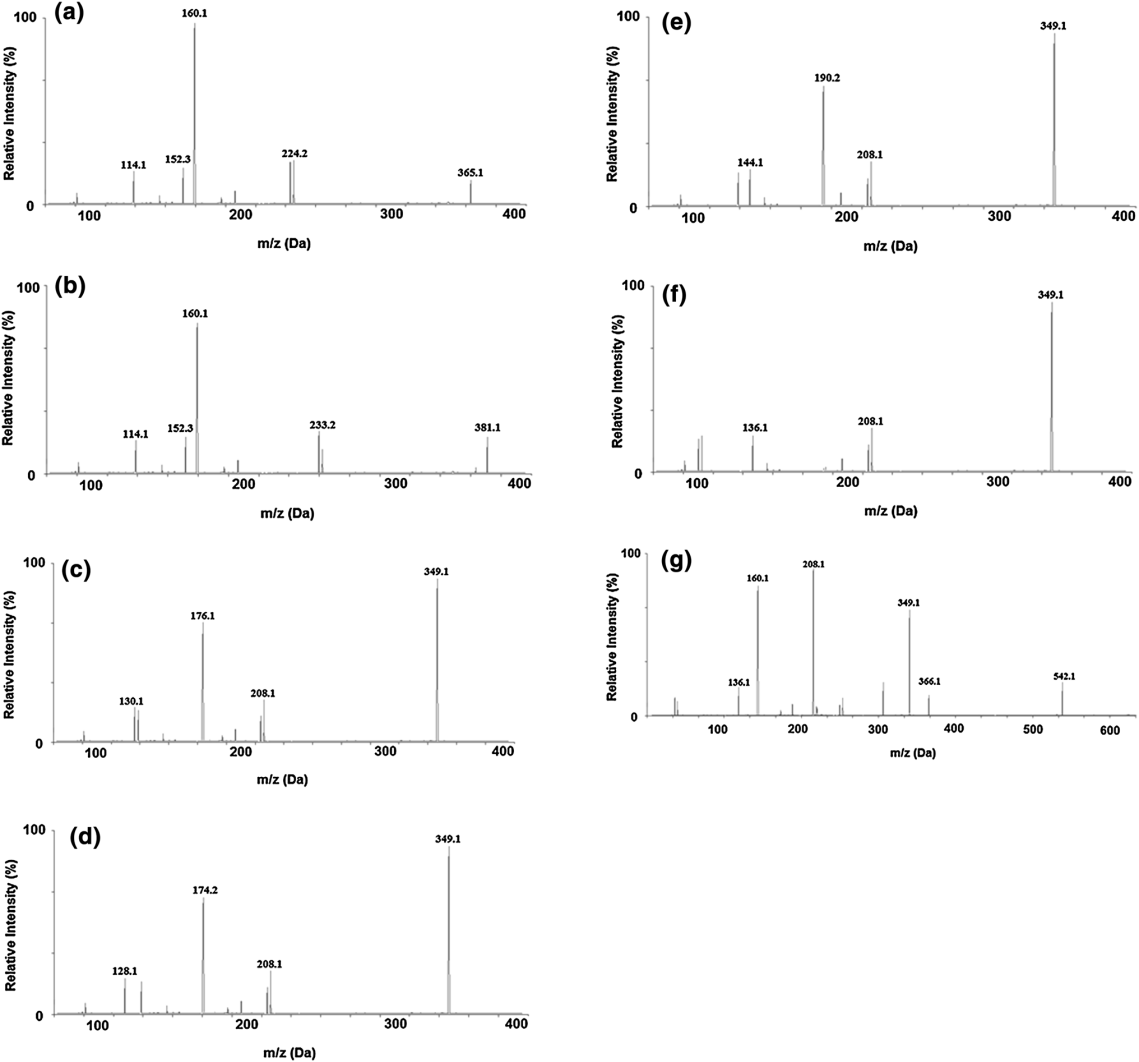
The representative product ion spectra corresponding to the potential metabolites M1–M7 (**a**–**g**)

The characteristic molecular ion of M1 (retention time 2.34 min) was obviously at *m*/*z* = 382 which was 16 Da lower than the protonated ion of AMOX, indicating that it was formed through hydroxylation of amoxicillin in aromatic moiety. Nevertheless, the software applied did not indicate the exact hydroxylation site. The components in the HLM extracts were identified and confirmed according to the mass spectrometric fragmentation mechanism through the means of MS/MS characteristic fragment ions. Figures 4 and 5 show MS/MS spectra of compounds M1–M7. The compound fragments easily yielded dominant characteristic ions, which indicates the presence of the relevant groups in the molecule.

Finally, the principal metabolic reactions for amoxicillin detected in vitro were oxidation, hydroxylation, oxidative deamination and conjugation with glucuronic acid. Spectra interpretation for compounds M1–M7 was carried out to identify suspect biotransformation products, and all fragmentations were analyzed thoroughly to draw general conclusions on fragmentation characteristics of potential metabolites, with the aim to help in and facilitate the identification of novel compounds for personalized antibiotic therapy. The metabolites detected after incubation with HLM were not found in the control samples. All phase I metabolic reactions found in this study are typically catalyzed by the P450 superfamily of enzymes [17].

### Method Validation

The multiple reaction monitoring mode was used to monitor the precursor ion and the corresponding product ion, which could reduce interference and enhance selectivity. Amoxicillin was detected at *m*/*z* 366 [M+H]^+^. Quantification was performed using MRM of the *m*/*z* 366 → 160 transition. The knowledge of such transition can be of interest to confirm the quantitative results obtained for the measurements of the target analyte in complex matrix mainly in the presence of interfering compounds.

Nine concentrations (1, 3, 5, 7, 10, 15, 20, 30 and 50 μg mL^−1^) defined the calibration curves. The calibration curve for amoxicillin was linear over the concentration range 1–50 μg mL^−1^. Standard curve used in this study was determined using the following linear regression: *y* = *ax* + *b* (SD_*xy*_ = 552, *R* ^2^ = 0.999), where *y* is the peak area, *a* (10,627, SD_*a*_ = 67) is the slope, *x* is the respective concentration and *b* (−1,480, SD_*b*_ = 308) is the intercept. The limit of detection (LOD) and limit of quantification (LOQ) were found to be 0.031 and 0.093 μg mL^−1^, respectively. Relative standard deviation (RSD) was 0.93 %.

Calibration curves of the human samples spiked with target compound was determined in the relevant concentration range. Obtained data with back calculated values, accuracy and precision are shown in Tables 2 and 3. The analysis of AMOX using MRM function was highly selective with no interfering compounds. Specificity was performed using six different lots of human liver microsomes.

**Table 2:** Intraday accuracy and precision of amoxicillin in human liver microsomes

Compound	Theoretical concentration (mg ml^−1^)	Observed concentration (mean ± SD)	Accuracy (%)	RSD
Amoxicillin (*n* = 10)	1	1.1 ± 0.3	108.3	6.83
	3	3.3 ± 0.01	107.7	1.66
	5	5.3 ± 0.7	105.5	6.25
	7	6.9 ± 0.4	98.1	0.24
	10	9.5 ± 0.4	94.8	3.10
	15	15.2 ± 0.3	101.1	3.26
	20	20.1 ± 0.4	100.4	2.44
	30	28.9 ± 0.1	96.2	2.90
	50	47.3 ± 0.6	94.4	2.76

**Table 3:** Accuracy and precision results

Nominal concentration (μg ml^−1^)		Intraday (*n* = 4)		Interday (*n* = 3)	
		%Bias	%CV	%Bias	%CV
LQC	1	−2.5	12.4	−10.7	12.6
MQC	20	−0.5	11.7	−5.0	7.9
HQC	50	−4.4	6.1	−6.9	5.6

Intra-assay and inter-assay accuracy and precision were assessed over three separate days by preparing a daily standard calibration curve along with 4 replicates of LQC, MQC, and HQC samples. Statistical analysis of the accuracy and precision is presented in Table 3. The intraday and interday %CV were ≤12.4 and ≤12.6 %, respectively. The intraday and interday %Bias was within ±4.4 and ±10.7 %, respectively.

Stability of AMOX in biological samples was tested at three concentration levels (1, 7 and 15 μg mL^−1^) under different conditions. The results of stability tests for biological samples: autosampler (accuracy ranging from −3.94 to 2.82 % and precision <1.21 % for the analyzed drug), freeze and thaw (accuracy ranging from −3.28 to 2.41 % and precision <4.21 % for the analyzed drug), short term (accuracy ranging from −5.21 to 4.38 % and precision <2.11 % for the analyzed drug), and preliminary long term at two storage temperatures (accuracy ranging from −5.92 to 4.35 % and precision <0.94 % for the analyzed drug). For each concentration level, there were acceptance criteria falling within the range 85–115 %. In addition, evaluated were the respective stability tests that confirmed the stability of amoxicillin in the stock and working solutions at 1–4 °C after 1 month. The target compound was proven to be stable both in stock solution (at −4 °C) and in human samples (at −20 °C) after long-term storage (for 1 month). These data indicated the analyte was stable during sample preparation and chromatographic measurements.

## Conclusions

A LC/MS method for the analysis of the amoxicillin as a model compound was developed and applied to obtain the first data regarding the chemical structures of its main phase I and phase II metabolites after incubation with human liver microsomes in vitro. The parent drug was incubated with human liver fractions to search for phase I metabolites. Thereafter, glucuronidation was also investigated. Elemental compositions of both the precursor and product ions were calculated based on mass measurements. Chemical structures of the metabolites detected were then proposed. Six phase I and one phase II metabolites were detected and structurally described. The metabolism of AMOX occurred via oxidation, hydroxylation and oxidative deamination, as well as the combination of these reactions. Compound M7, with glucuronic acid was also observed as phase II metabolite. Neither sulfate nor glutathione conjugates were detected. Proposed analytical procedure was shown to be adequate for the intended pharmacokinetic applications, i.e., the incubations experiments with microsomes originating from liver samples from patients underwent antibacterial therapy. Additionally, although the total level of liver microsomal P450s does not vary considerably among humans, genetic polymorphism and susceptibility to induction of a given P450 isoenzyme result in interindividual variations in levels of P450 isoforms (CYPs) [18]. In this light, all data concerning selectivity in catalytic properties of various CYPs towards a potent drug are desirable [19]. The above-discussed possibilities of selective metabolism of AMOX presumably dependent on the expression of P450 isoenzymes build up a background of individualized direct therapy with this drug, which is particularly critical in the case of antibiotic therapy.
